# Supplementary figures and images for: Reverse-engineered models reveal differential membrane properties of autonomic and cutaneous unmyelinated fibers
Source: PLoS Comput Biol. 2024 Oct 7;20(10):e1012475. doi: 10.1371/journal.pcbi.1012475 (PMC11486378; doi:10.1371/journal.pcbi.1012475)

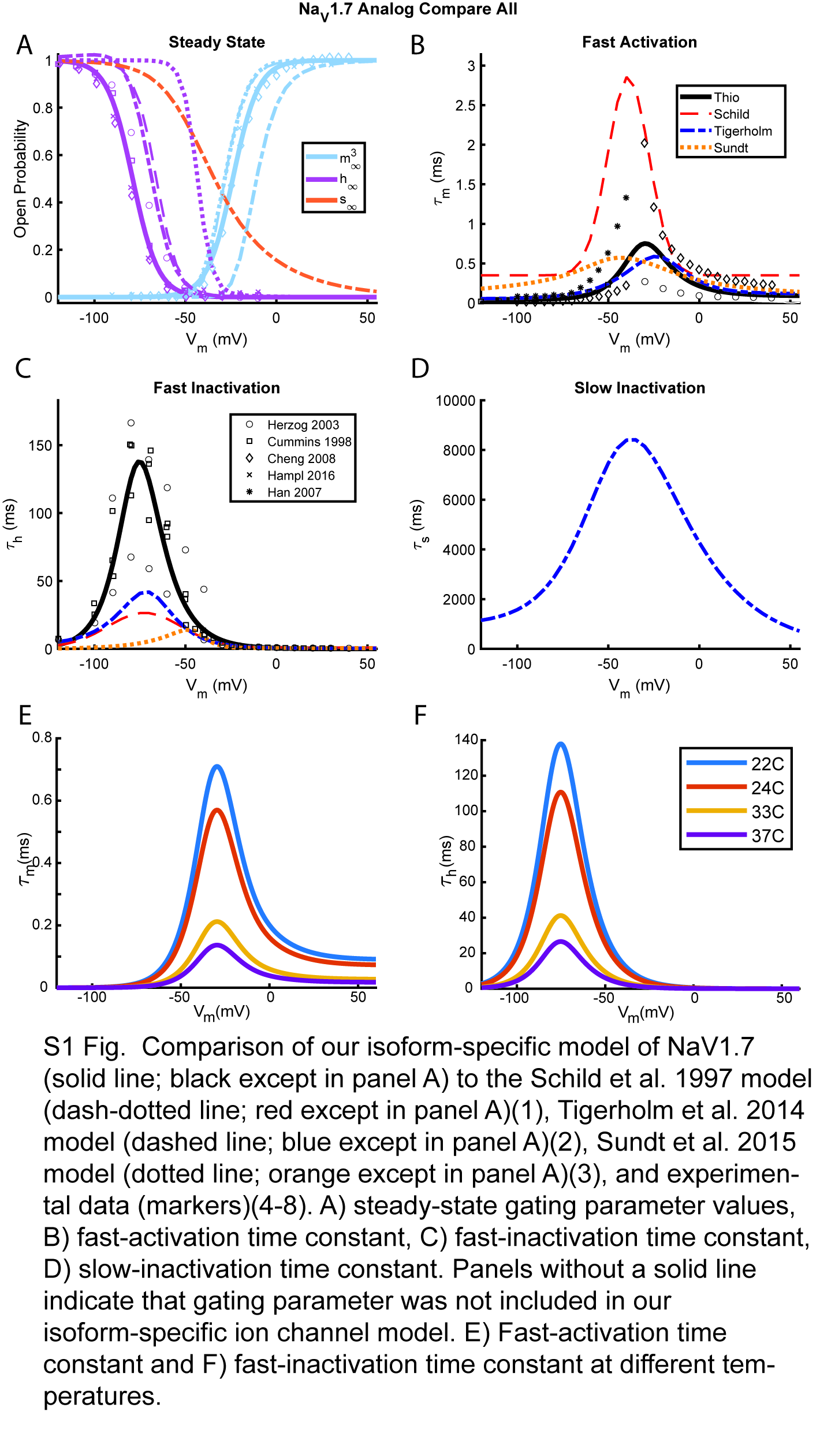

Supplement: S1 Fig — Comparison of our isoform-specific model of NaV1.7 (solid line; black except in panel A) to the Schild et al. 1997 model (dash-dotted line; red except in panel A) [14], Tigerholm et al. 2014 model (dashed line; blue except in panel A) [11], Sundt et al. 2015 model (dotted line; orange except in panel A) [12], and experimental data (markers) [57–61]. A) steady-state gating parameter values, B) fast-activation time constant, C) fast-inactivation time constant, D) slow-inactivation time constant. Panels without a solid line indicate that gating parameter was not included in our isoform-specific ion channel model. E) Fast-activation time constant and F) fast-inactivation time constant at different temperatures. (TIF) [file pcbi.1012475.s004.tif]

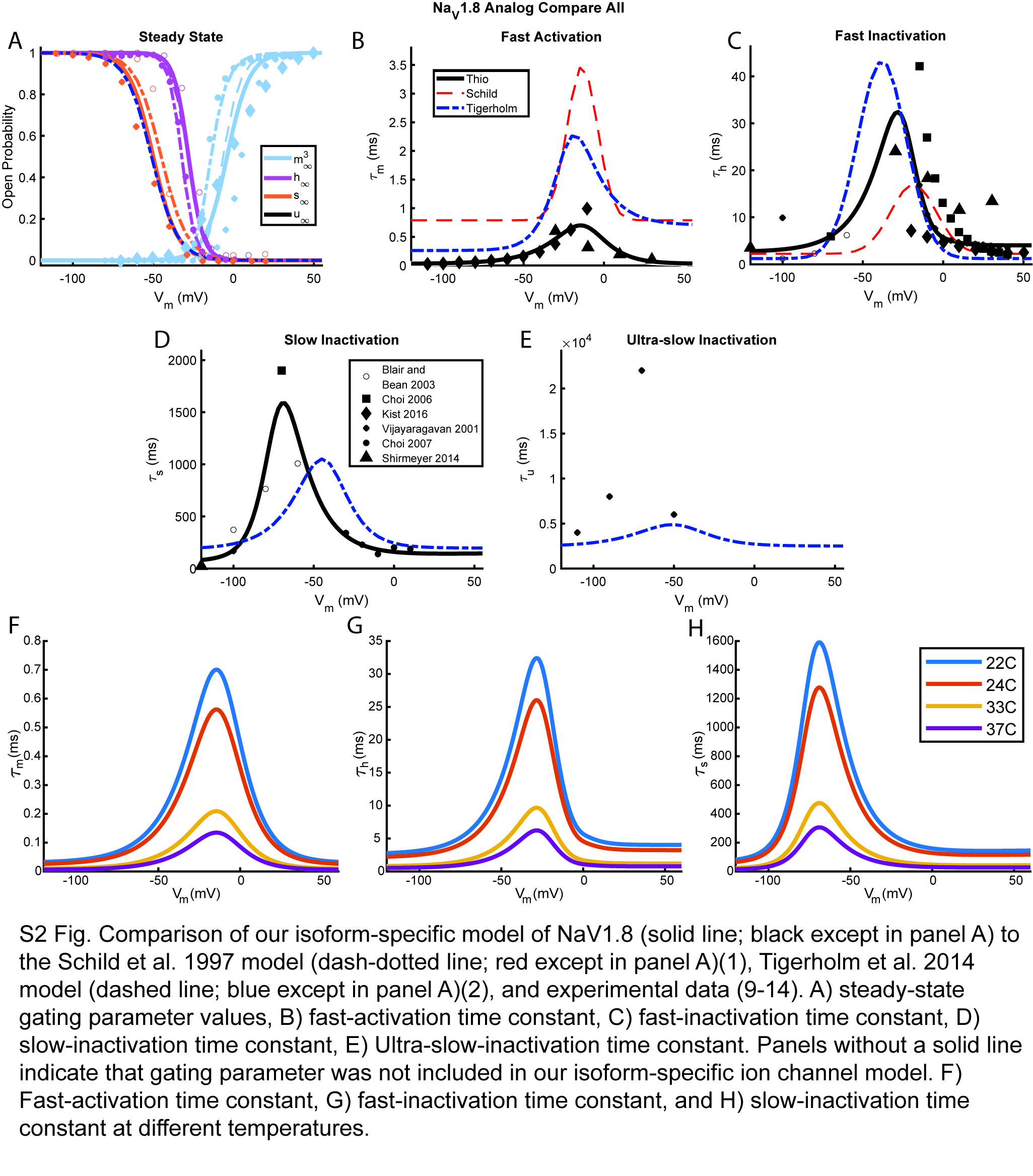

Supplement: S2 Fig — Comparison of our isoform-specific model of NaV1.8 (solid line; black except in panel A) to the Schild et al. 1997 model (dash-dotted line; red except in panel A) [14], Tigerholm et al. 2014 model (dashed line; blue except in panel A) [11], and experimental data [17, 18, 20, 21, 62, 63]. A) steady-state gating parameter values, B) fast-activation time constant, C) fast-inactivation time constant, D) slow-inactivation time constant, E) Ultra-slow-inactivation time constant. Panels without a solid line indicate that gating parameter was not included in our isoform-specific ion channel model. F) Fast-activation time constant, G) fast-inactivation time constant, and H) slow-inactivation time constant at different temperatures. (TIF) [file pcbi.1012475.s005.tif]

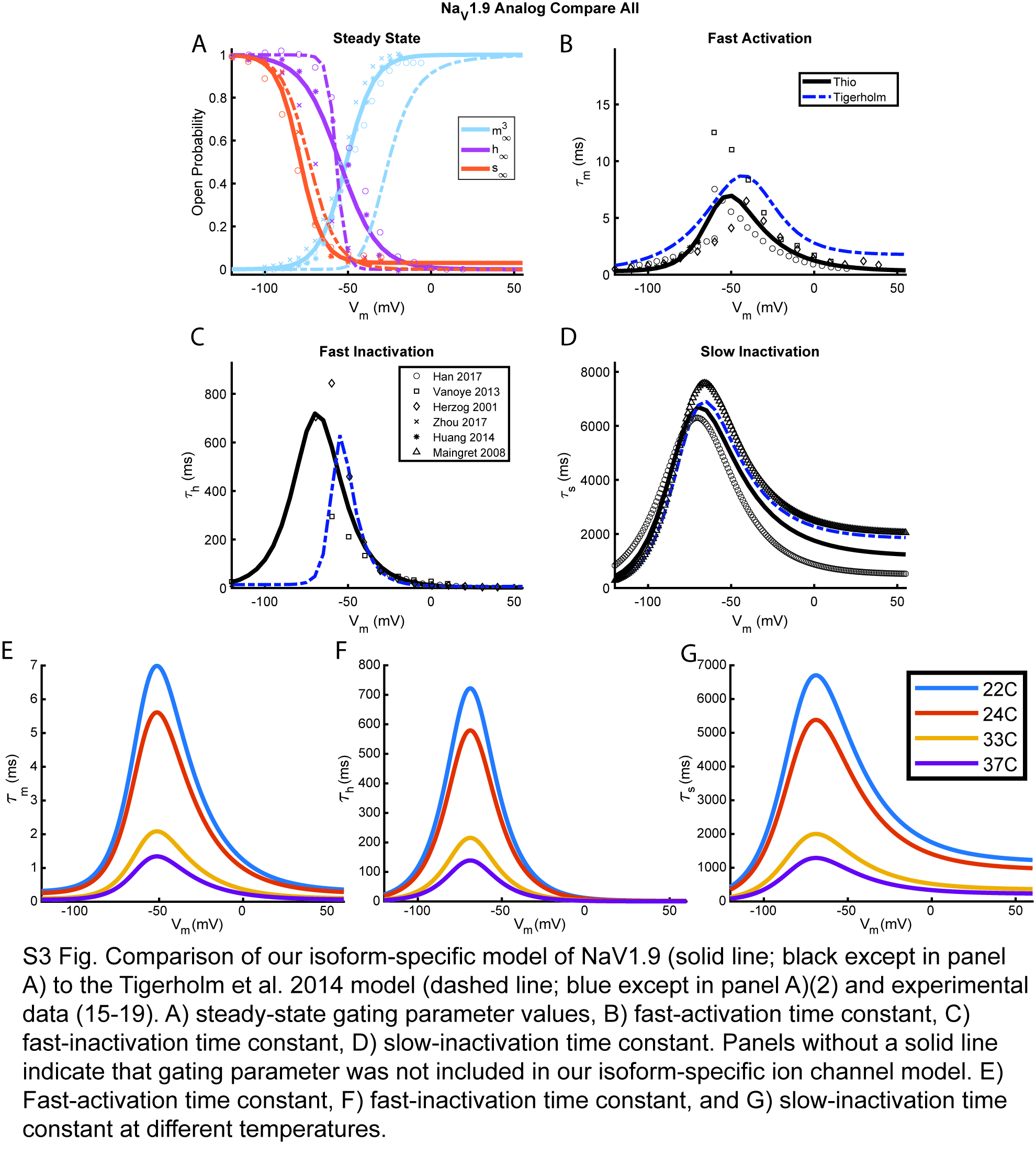

Supplement: S3 Fig — Comparison of our isoform-specific model of NaV1.9 (solid line; black except in panel A) to the Tigerholm et al. 2014 model (dashed line; blue except in panel A) [11] and experimental data [64–68]. A) steady-state gating parameter values, B) fast-activation time constant, C) fast-inactivation time constant, D) slow-inactivation time constant. Panels without a solid line indicate that gating parameter was not included in our isoform-specific ion channel model. E) Fast-activation time constant, F) fast-inactivation time constant, and G) slow-inactivation time constant at different temperatures. (TIF) [file pcbi.1012475.s006.tif]

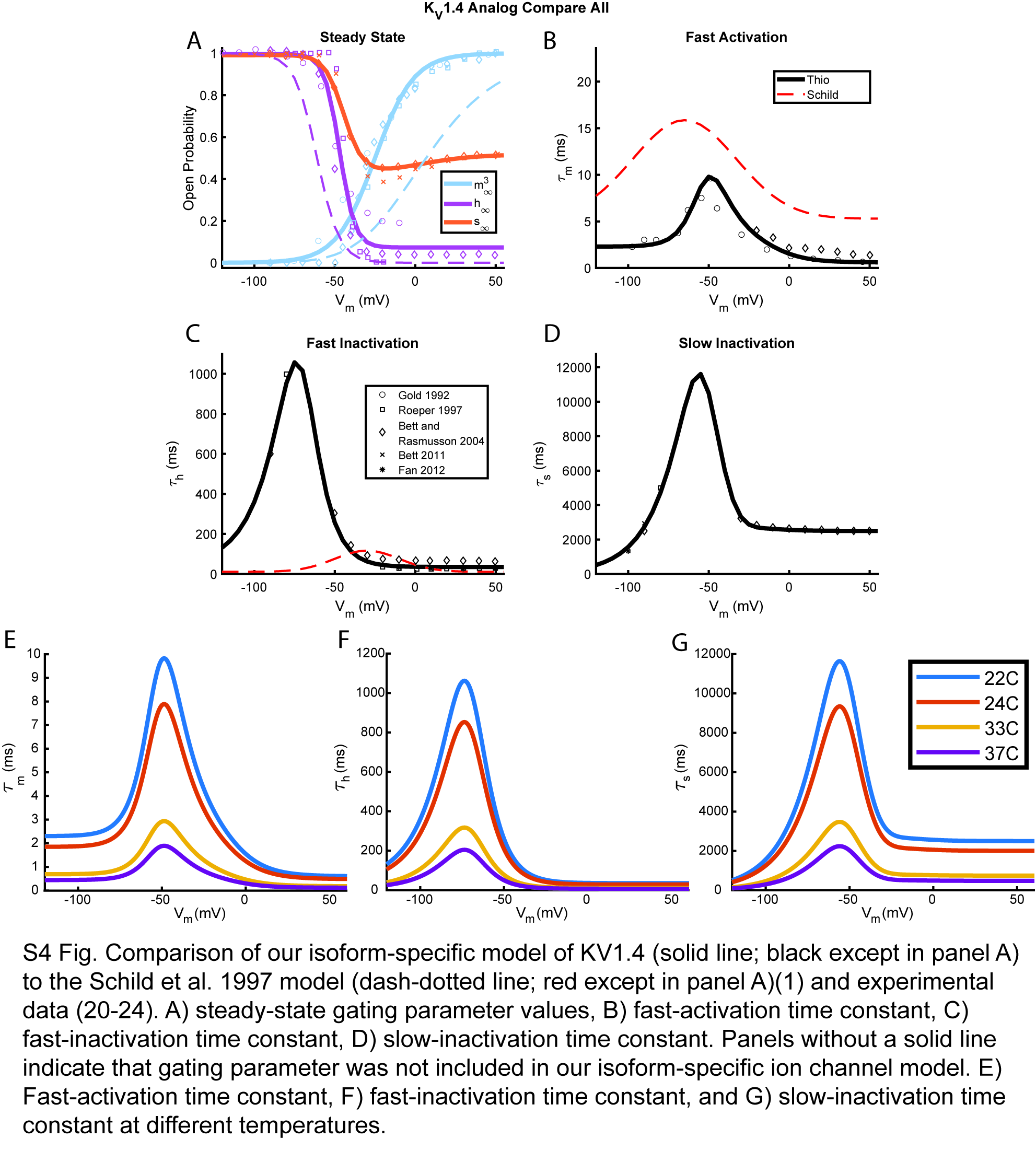

Supplement: S4 Fig — Comparison of our isoform-specific model of KV1.4 (solid line; black except in panel A) to the Schild et al. 1997 model (dash-dotted line; red except in panel A) [14] and experimental data [69–73]. A) steady-state gating parameter values, B) fast-activation time constant, C) fast-inactivation time constant, D) slow-inactivation time constant. Panels without a solid line indicate that gating parameter was not included in our isoform-specific ion channel model. E) Fast-activation time constant, F) fast-inactivation time constant, and G) slow-inactivation time constant at different temperatures. (TIF) [file pcbi.1012475.s007.tif]

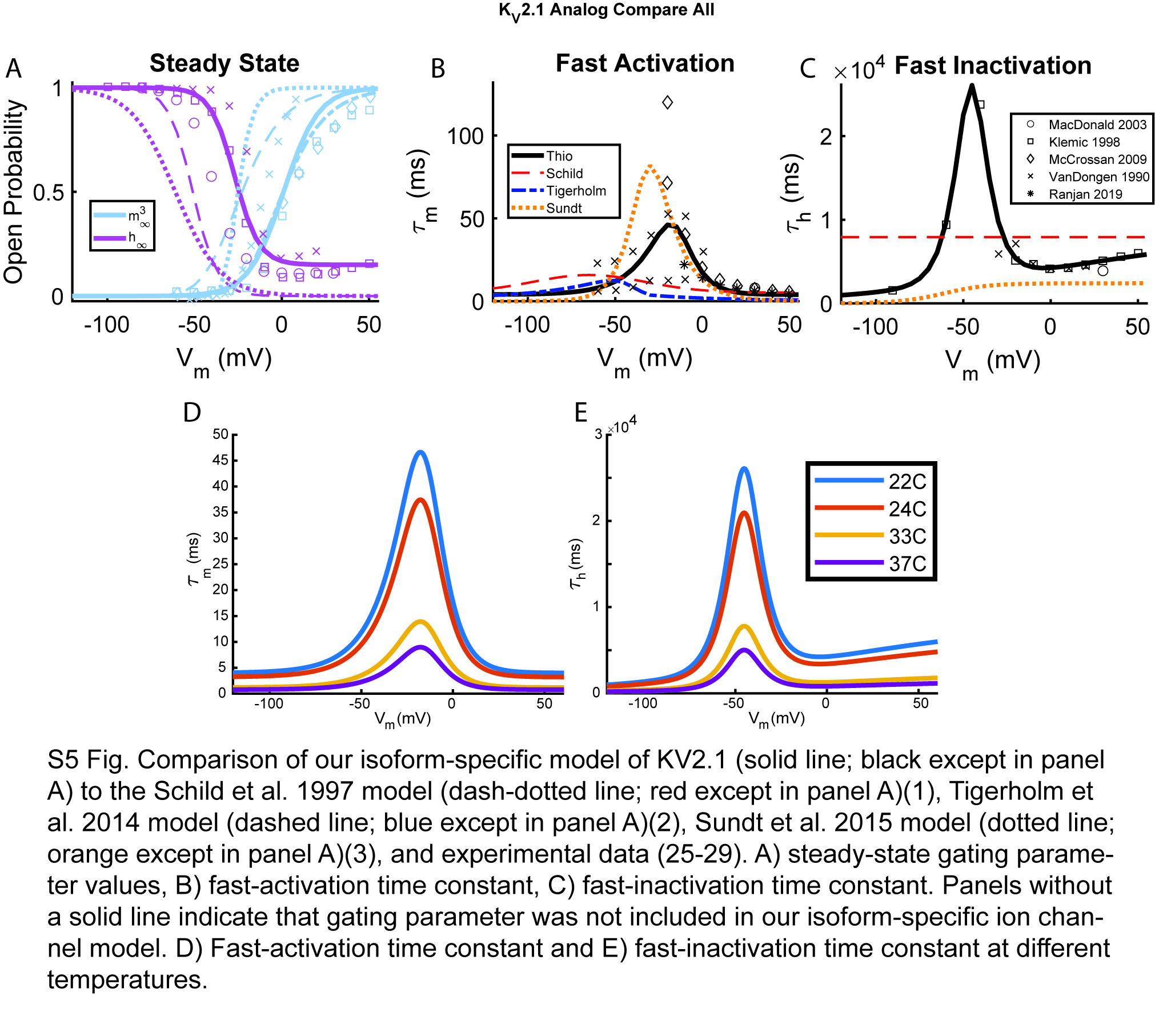

Supplement: S5 Fig — Comparison of our isoform-specific model of KV2.1 (solid line; black except in panel A) to the Schild et al. 1997 model (dash-dotted line; red except in panel A) [14], Tigerholm et al. 2014 model (dashed line; blue except in panel A) [11], Sundt et al. 2015 model (dotted line; orange except in panel A) [12], and experimental data [74–78]. A) steady-state gating parameter values, B) fast-activation time constant, C) fast-inactivation time constant. Panels without a solid line indicate that gating parameter was not included in our isoform-specific ion channel model. D) Fast-activation time constant and E) fast-inactivation time constant at different temperatures. (TIF) [file pcbi.1012475.s008.tif]

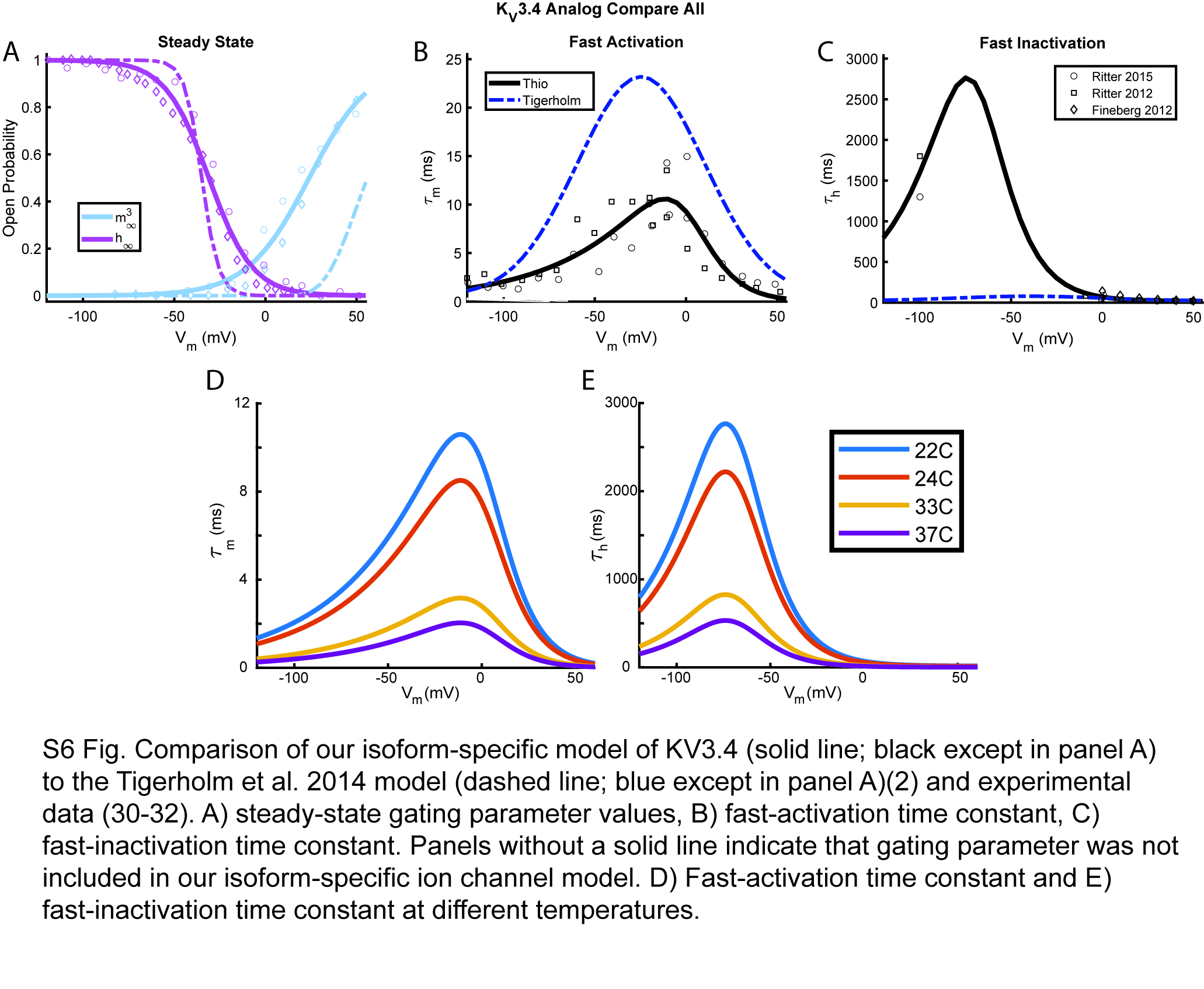

Supplement: S6 Fig — Comparison of our isoform-specific model of KV3.4 (solid line; black except in panel A) to the Tigerholm et al. 2014 model (dashed line; blue except in panel A) [11] and experimental data [79–81]. A) steady-state gating parameter values, B) fast-activation time constant, C) fast-inactivation time constant. Panels without a solid line indicate that gating parameter was not included in our isoform-specific ion channel model. D) Fast-activation time constant and E) fast-inactivation time constant at different temperatures. (TIF) [file pcbi.1012475.s009.tif]

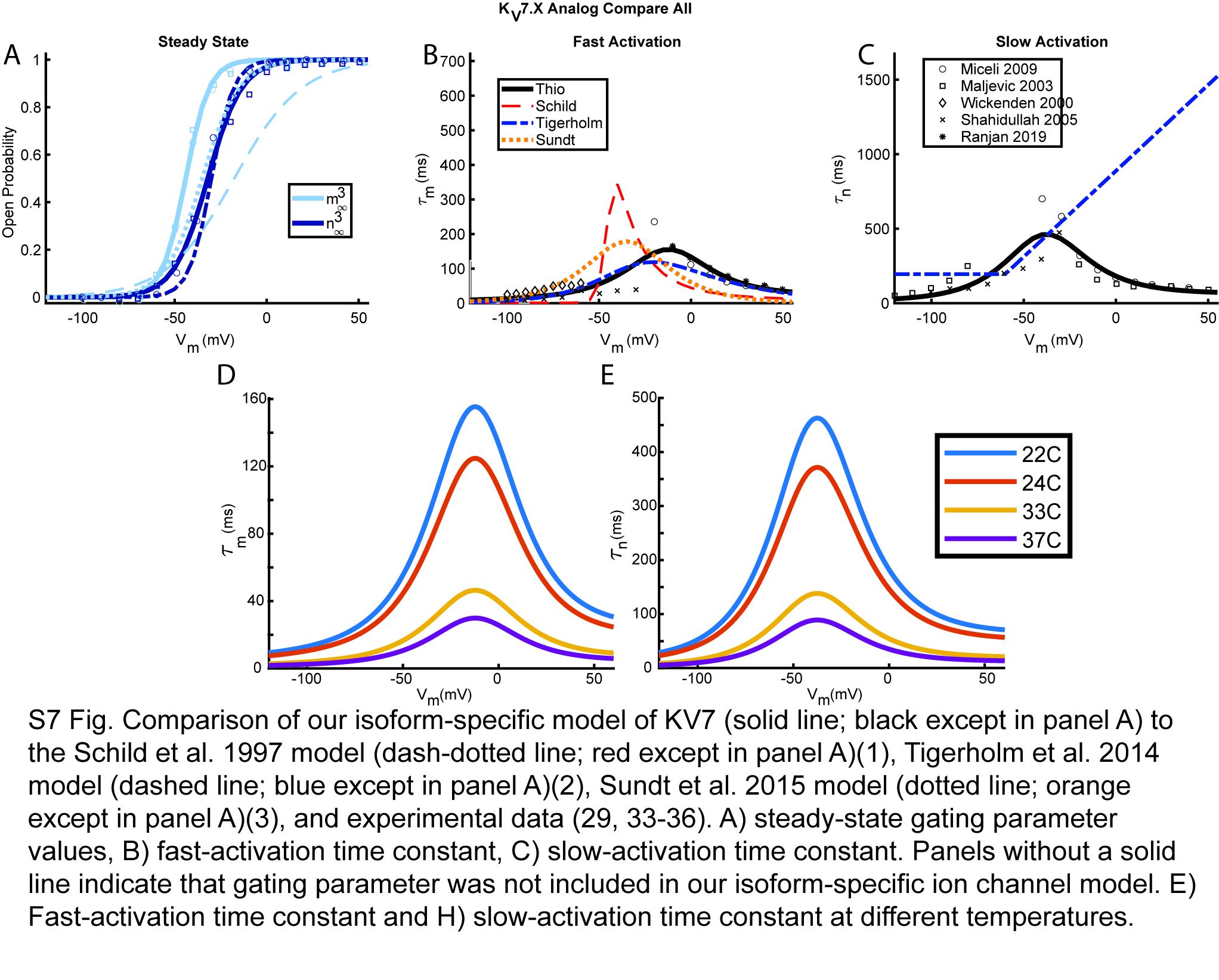

Supplement: S7 Fig — Comparison of our isoform-specific model of KV7 (solid line; black except in panel A) to the Schild et al. 1997 model (dash-dotted line; red except in panel A) [14], Tigerholm et al. 2014 model (dashed line; blue except in panel A) [11], Sundt et al. 2015 model (dotted line; orange except in panel A) [12], and experimental data [78, 82–85]. A) steady-state gating parameter values, B) fast-activation time constant, C) slow-activation time constant. Panels without a solid line indicate that gating parameter was not included in our isoform-specific ion channel model. E) Fast-activation time constant and H) slow-activation time constant at different temperatures. (TIF) [file pcbi.1012475.s010.tif]

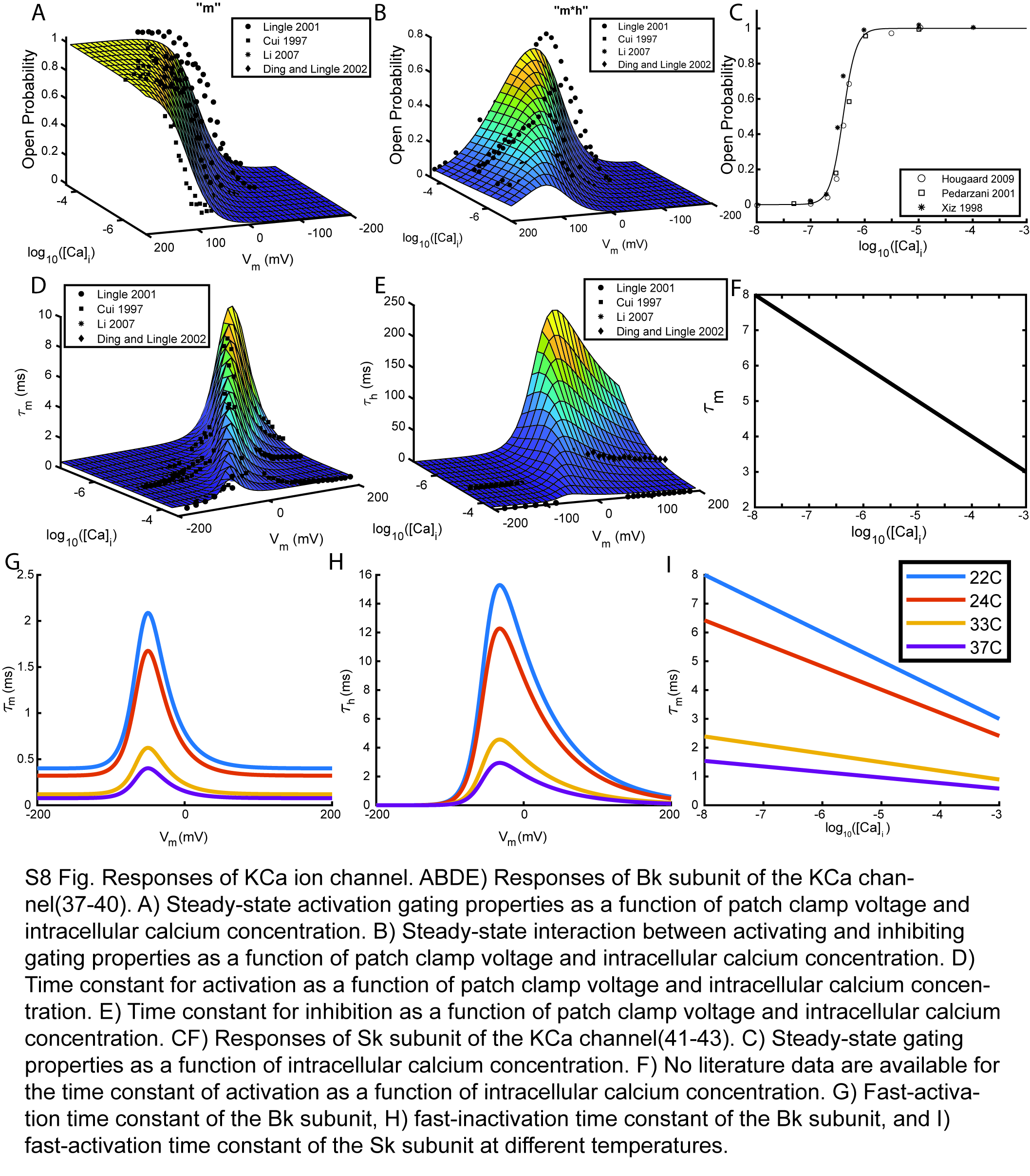

Supplement: S8 Fig — ABDE) Responses of Bk subunit of the KCa channel [86–89]. A) Steady-state activation gating properties as a function of patch clamp voltage and intracellular calcium concentration. B) Steady-state interaction between activating and inhibiting gating properties as a function of patch clamp voltage and intracellular calcium concentration. D) Time constant for activation as a function of patch clamp voltage and intracellular calcium concentration. E) Time constant for inhibition as a function of patch clamp voltage and intracellular calcium concentration. CF) Responses of Sk subunit of the KCa channel [90–92]. C) Steady-state gating properties as a function of intracellular calcium concentration. F) No literature data are available for the time constant of activation as a function of intracellular calcium concentration. G) Fast-activation time constant of the Bk subunit, H) fast-inactivation time constant of the Bk subunit, and I) fast-activation time constant of the Sk subunit at different temperatures. (TIF) [file pcbi.1012475.s011.tif]

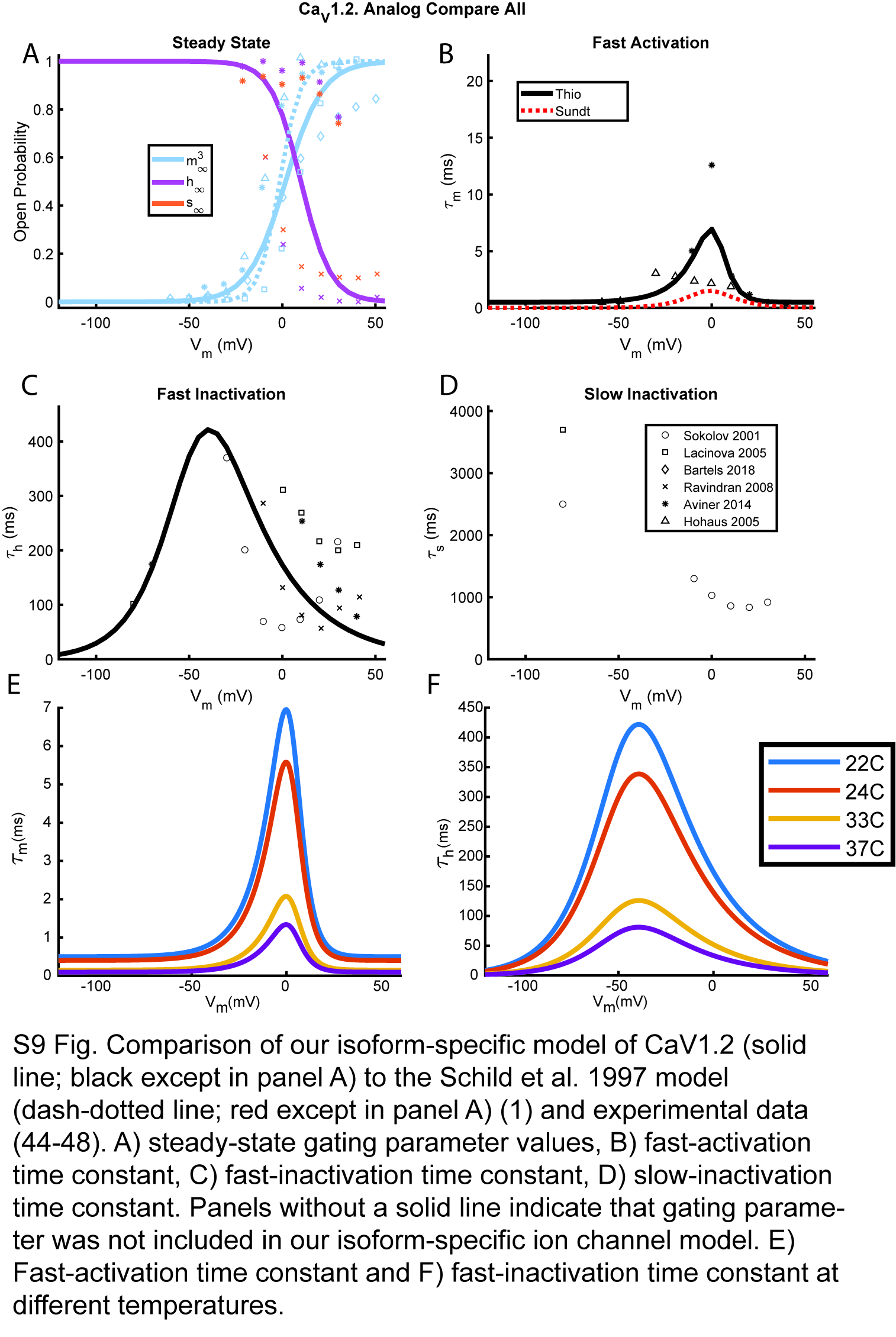

Supplement: S9 Fig — Comparison of our isoform-specific model of CaV1.2 (solid line; black except in panel A) to the Schild et al. 1997 model (dash-dotted line; red except in panel A) [14] and experimental data [93–97]. A) steady-state gating parameter values, B) fast-activation time constant, C) fast-inactivation time constant, D) slow-inactivation time constant. Panels without a solid line indicate that gating parameter was not included in our isoform-specific ion channel model. E) Fast-activation time constant and F) fast-inactivation time constant at different temperatures. (TIF) [file pcbi.1012475.s012.tif]

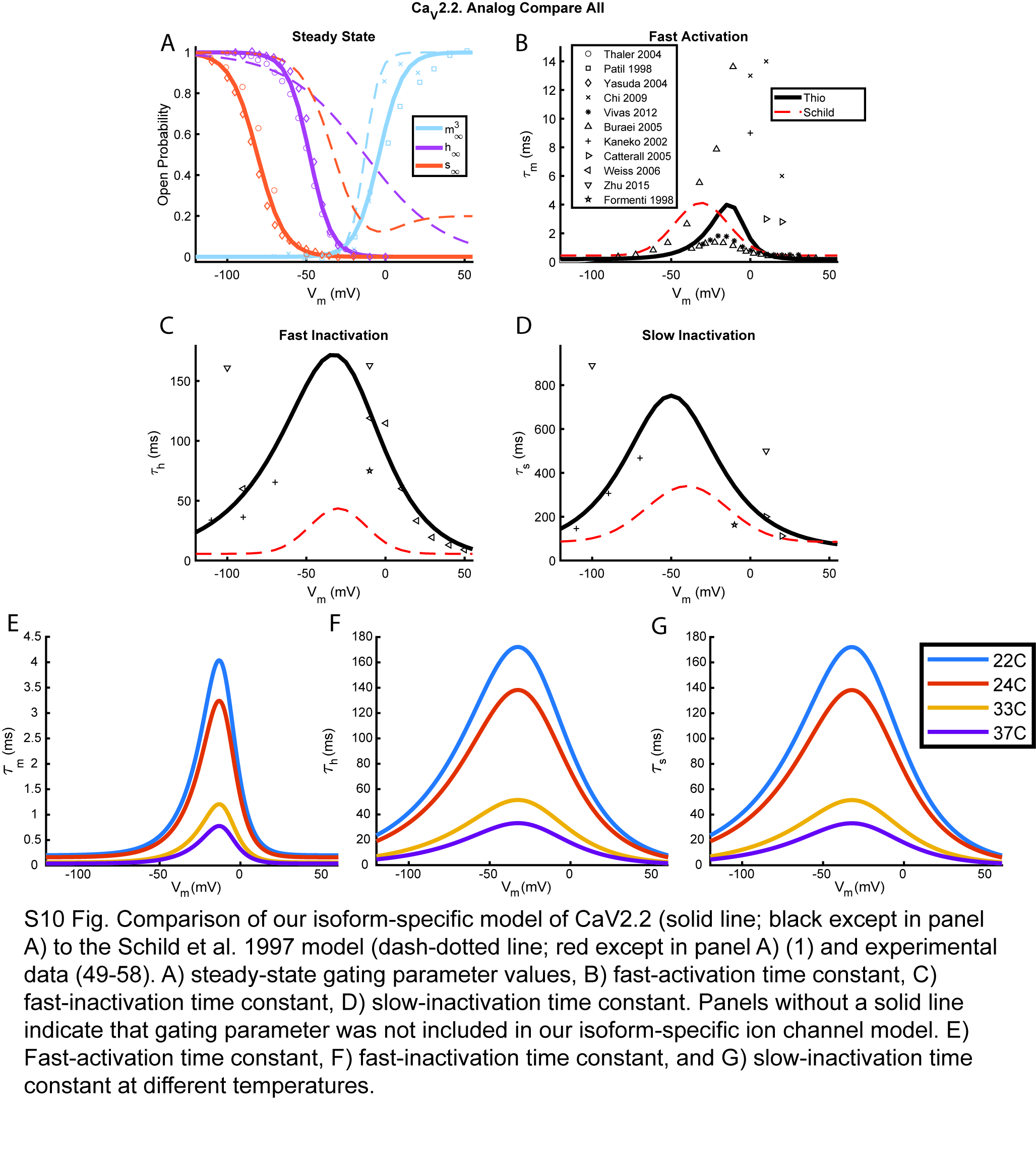

Supplement: S10 Fig — Comparison of our isoform-specific model of CaV2.2 (solid line; black except in panel A) to the Schild et al. 1997 model (dash-dotted line; red except in panel A) [14] and experimental data [98–107]. A) steady-state gating parameter values, B) fast-activation time constant, C) fast-inactivation time constant, D) slow-inactivation time constant. Panels without a solid line indicate that gating parameter was not included in our isoform-specific ion channel model. E) Fast-activation time constant, F) fast-inactivation time constant, and G) slow-inactivation time constant at different temperatures. (TIF) [file pcbi.1012475.s013.tif]

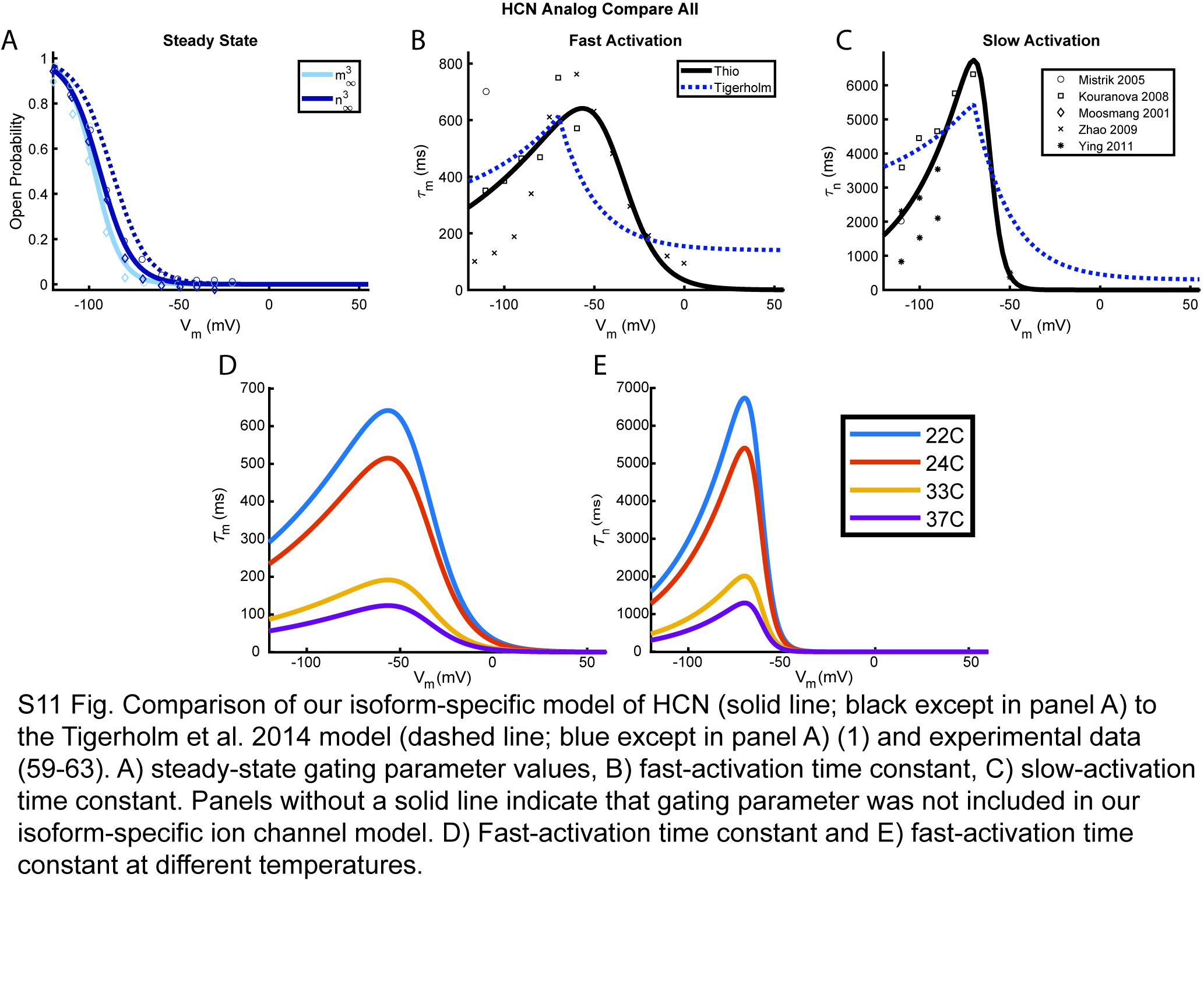

Supplement: S11 Fig — Comparison of our isoform-specific model of HCN (solid line; black except in panel A) to the Tigerholm et al. 2014 model (dashed line; blue except in panel A) [14] and experimental data [108–112]. A) steady-state gating parameter values, B) fast-activation time constant, C) slow-activation time constant. Panels without a solid line indicate that gating parameter was not included in our isoform-specific ion channel model. D) Fast-activation time constant and E) fast-activation time constant at different temperatures. (TIF) [file pcbi.1012475.s014.tif]

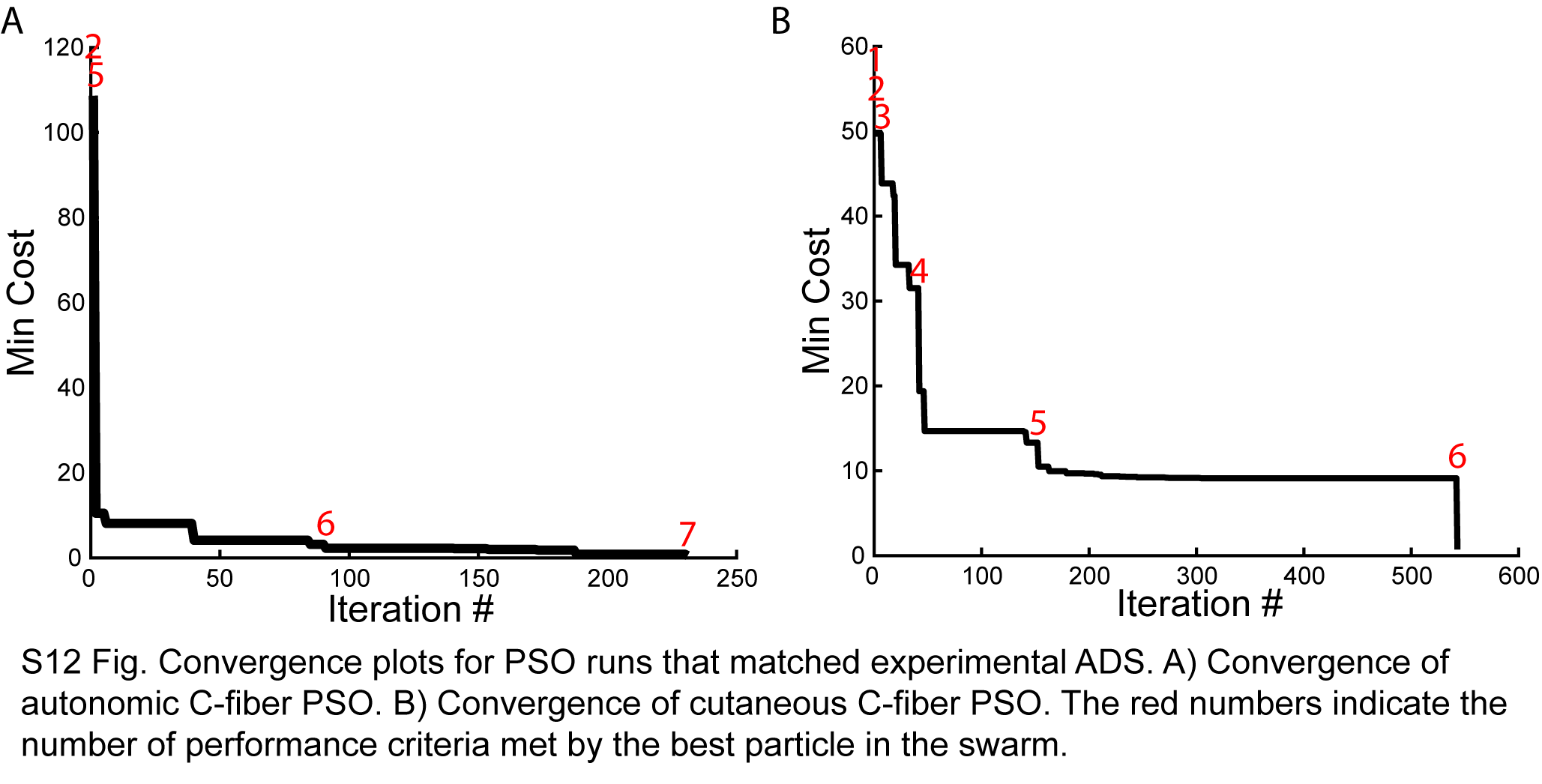

Supplement: S12 Fig — A) Convergence of autonomic C-fiber PSO. B) Convergence of cutaneous C-fiber PSO. The red numbers indicate the number of performance criteria met by the best particle in the swarm. (TIF) [file pcbi.1012475.s015.tif]

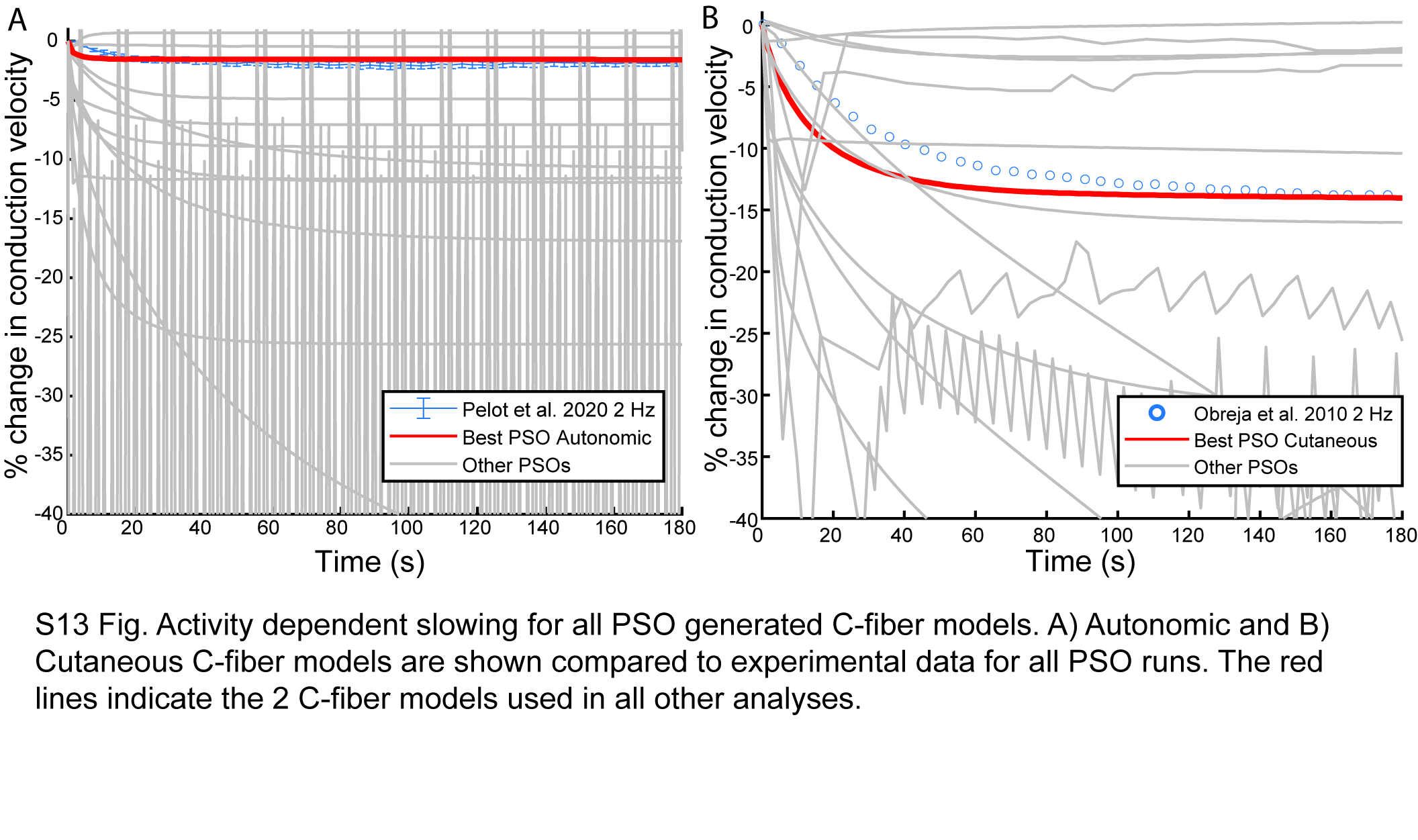

Supplement: S13 Fig — A) Autonomic and B) Cutaneous C-fiber models are shown compared to experimental data for all PSO runs. The red lines indicate the 2 C-fiber models used in all other analyses. (TIF) [file pcbi.1012475.s016.tif]

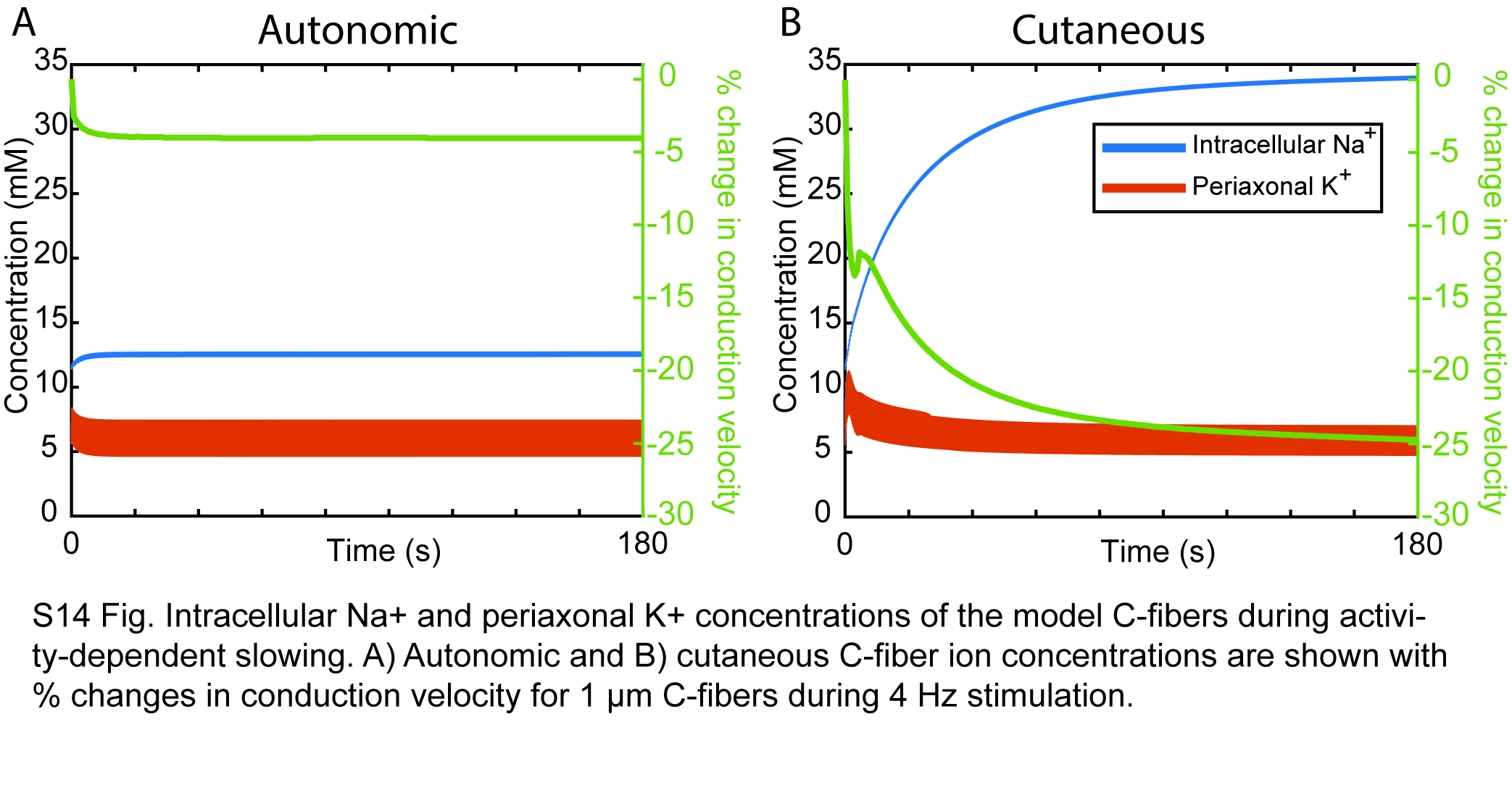

Supplement: S14 Fig — A) Autonomic and B) cutaneous C-fiber ion concentrations are shown with % changes in conduction velocity for 1 μm C-fibers during 4 Hz stimulation. (TIF) [file pcbi.1012475.s017.tif]
