# Supplementary material for: Reverse-engineered models reveal differential membrane properties of autonomic and cutaneous unmyelinated fibers
Source: PLoS Comput Biol. 2024 Oct 7;20(10):e1012475. doi: 10.1371/journal.pcbi.1012475 (PMC11486378; doi:10.1371/journal.pcbi.1012475)
Supplement: Attachment — Submitted filename: PCOMPBIOL-D-24-00176_ResponseToReviewers.docx [file pcbi.1012475.s018.docx]

PCOMPBIOL-D-24-00176

"Reverse-engineered models reveal differential membrane properties of autonomic and cutaneous unmyelinated fibers"

**Response to Reviewers**

We thank the Reviewers for their insightful comments and critical review of our work. We revised the manuscript to address the concerns, as detailed below.

**Reviewer 1**

1) Please, briefly define chronaxie when it first appears

We defined the chronaxie in the revised manuscript, including in the Methods:

*“We defined the target range for chronaxie from 750 to 1500 µs for the autonomic fiber (26, 27) and from 500 to 1500 µs for the cutaneous fiber (28, 29). The chronaxie is the shortest pulse width that can evoke an action potential with twice the rheobase current, where the rheobase is the minimum current needed to evoke an action potential with a pulse of infinite duration.”*

and in the Results:

*“Both models exhibited a >10% change in conduction velocity when we changed Na_V_1.8 and Ra, in chronaxie (minimum pulse width that can evoke an action potential with twice the minimum activation current) when we changed Na_V_1.7 and Na_V_1.8, and in action potential duration when we changed Na_V_1.8 and K_V_2.1 (Fig 6a-c and Fig 7a-c).”*

2) A few panels are empty in most of Supplementary Figures. Please correct or delete them.

We deleted the empty panels in Supplementary Figures 1-11.

**Reviewer 2**

3) It is not clear how the authors chose a set of voltage-gated channels on the C-fibers. Do all channels have a rationale with immunohistochemistry or related morphological studies? Is it rational to include the channels with no evidence for their existence? What is an assumption on the passive membrane properties and intracellular environment? How they defined periaxonal space? At least, the authors need to thorough description of these points.

We agree with the Reviewer that it is important to make clearer the rationale for model parameters, and we clarified these choices in the Methods of the revised manuscript.

We only included voltage-gated ion channel isoforms that either were present in previous C-fiber axon models or were shown, in experimental characterization, to be present in peripheral C-fiber axons (Pelot et al. 2021).

We assumed the membrane capacitance was ~1.33 µF/cm^2^, which is consistent with previous C-fiber models (Schild et al. 1994). The PSO optimized the intracellular resistance and the balance function to control the passive membrane properties.

We defined the periaxonal space as the 30 nm surrounding the axon, which is consistent with SEM imaging (Dimitrov 2005). We assumed that the difference in ion concentration between the periaxonal space and intracellular environment determined the dynamic reversal potential of each ion. We then allowed ions to diffuse between the periaxonal space and the extracellular environment which had constant ion concentrations.

We added the following text to clarify these points:

“*We developed models of specific ion channel isoforms found in unmyelinated peripheral neurons (3) using voltage clamp data from multiple publications.*”

“*We used multi-objective particle swarm optimization to determine 17 parameters of our 1 µm C-fiber models: the maximum conductances of the 12 ion channels, maximum currents of the NaK pump and NaCa exchanger, intracellular resistance, rest potential, and the total conductance and pump current (Fig 8b). We assumed the membrane capacitance was ~1.33 µF/cm^2^ (13) and leakage currents were controlled by the balance function.*”

“*Both accumulation mechanisms allowed diffusion between the periaxonal space (30 nm thick (46)) and an extracellular bath with constant ion concentrations.*”

Pelot, N. A., Catherall, D. C., Thio, B. J., Titus, N. D., Liang, E. D., Henriquez, C. S., & Grill, W. M. (2021). Excitation properties of computational models of unmyelinated peripheral axons. *Journal of neurophysiology*, *125*(1), 86-104.

Schild, J. H., Clark, J. W., Hay, M., Mendelowitz, D., Andresen, M. C., & Kunze, D. L. (1994). A-and C-type rat nodose sensory neurons: model interpretations of dynamic discharge characteristics. *Journal of neurophysiology*, *71*(6), 2338-2358.

Dimitrov, A. G. (2005). Internodal sodium channels ensure active processes under myelin manifesting in depolarizing afterpotentials. *Journal of theoretical biology*, *235*(4), 451-462.

4) It is also not clear whether they assume a uniform distribution of the set of channels for the whole courses of the axons. It is well known that distribution is not uniform, i.e. highest Na conductances at the proximal axons near the physiological spike initiation sites.

We agree that the ion channel densities are not uniform across the entire course of the axon where there are specifically more sodium ion channels in the axon initial section where action potentials initiate. However, we assumed a uniform distribution of the set of ion channels for the whole course of the axons because many of the applications of our C-fiber models (electrical nerve stimulation, pain transmission, spinal cord stimulation) interface with the axon at points far from the terminals and axon initial segment.

We added text to describe this distinction in the Discussion:

“*We also assumed a uniform distribution of each ion channel density along the length of the axon, which may not reflect differences in distributions near the axon initial segment or axon terminals. However, many of the applications of our C-fiber model interface with the axons where ion channel densities are likely more uniform (41).*”

5) In my opinion, testing in other models of the axon with experimental voltage-clamped data, such as the squid giant axon or hippocampal mossy fiber axon, is obligatory to show the rationality of this optimization approach.

We agree that we have not proven that our PSO approach can be used to generate realistic neuron models across the nervous system. However, our goal was to develop models of mammalian C-fiber axons, which are notoriously difficult to model because the axons are tiny and extremely difficult to characterize with patch clamp. Therefore, we used our PSO approach to develop two types of C-fiber axons with disparate conduction responses, autonomic and cutaneous C-fibers. We used the same fundamental ion channel models, which are present in both types of C-fiber axons and showed that different conduction responses seen in different populations of C-fiber axons can originate solely from differential ion channel expression levels. While this does not definitively prove that our PSO approach can be readily applied to other types of axon and neuron models, it does show that our approach can be used to develop a family of different C-fiber axon models, which was our goal.

We added this limitation and future opportunity in the Discussion.

“*While we developed two mammalian C-fiber models with our PSO, the methods can be extended to optimize objectively other types of neuron models. However, additional validation of the PSO must be conducted to ensure that the approach is sufficiently robust to model accurately other types of neurons.*”

**Reviewer 3**

6) All but two of the sources of experimental data for the ion channel properties were performed at or near room temperature (Table S1). It would be helpful in the Methods section to comment on how the temperature-dependence (Q10) of each of the channels was determined/arrived at and their validity used in the mod files. A supplemental figure illustrating the temperature dependence of the conductances/pumps on the voltage-gated currents’ kinetics would be instructive. Also, it is not clear how the PSO was applied between two different temperatures when optimizing action potential duration and intracellular current threshold whose target temperatures were near room temperature (Table 1).

We agree that the temperature dependence is an important factor in modeling ion channel dynamics. We chose to use a standard Q10 value of 3 for all ion channel models. While ion channels likely do not all have the same Q10 scaling factor, we were not able to identify data that rigorously quantified the temperatures scaling ion channel kinetics for all our modeled ion channel isoforms. Therefore, we chose a Q10 value that was consistent with Q10 values from previous modeling studies, which have used values from 1.5 to 4 (Schild et al. 1994, Schild et al. 1997, Tigerholm et al. 2014, and Sundt et al. 2015).

The PSO was able to apply different temperatures during optimization by separating each conduction response into a different set of simulations. The PSO then simulated tests for each conduction response at the relevant temperature based on the temperature at which the experimental data were gathered. This allowed us to run simulations at body temperature for conduction velocity and simulations at room temperature for intracellular threshold.

We added panels to Supplemental Figure 1-11 to show the scaling of ion channel kinetics as a function of temperature. We further clarified our standard Q10 factor in the Methods:

“*To account for the scaling of ion channel kinetics at different temperatures, we used a standard Q10 factor of 3, which is within the range (1.5 to 4) of Q10 factors used in previous C-fiber modeling studies* (11-14).”

We also added this limitation in the Discussion:

“*Finally, we chose to use a standard Q10 factor to scale ion channel kinetics across different temperatures because data rigorously characterizing the Q10 factor for all of our modeled ion channel isoforms were unavailable. However, different ion channel isoforms may have different Q10 scaling factors.*”

Methods

7) Supplemental Figures 1-11 are a bit difficult, especially in the context of demonstrating that they “accurately represent the available patch clamp data”. The work presented here might be better served by simply comparing their new channel models to the experimental data, rather than the channel models used in previous in silico studies. It also might be here that the temperature-dependence is illustrated.

We chose to include the previous in silico studies to emphasize the necessity of our rigorous ion channel modeling work given that previous ion channel models did not represent well the available experimental data. We believe that this is an important point and is the foundation of the differences in conduction responses seen in Figure 3, so we chose in include these data in Supplemental Figures 1-11. However, we did simplify these figures by omitting all empty panels to improve clarity.

We also added characterizations of the temperature dependence of our ion channel models to Supplemental Figures 1-11.

8) Table 1 the range of values determined in this study might be added to this table for comparison to the “Target” / experimentally measured ranges. And so might want to move this table to the results section.

We moved Table 1 to the Results.

9) Page 5, line 83: Please clarify the sentence “We selected isoforms such that each class of identifiable voltage-sensitive current in peripheral C-fibers was represented by at least one model ion channel.”

We clarified the sentence to indicate that we included only voltage-gated ion channel isoforms that were present in previous C-fiber axon models or were shown, in experimental characterization, to be present in peripheral C-fiber axons (Pelot et al. 2021):

“*We chose to only include voltage-gated ion channel isoforms that were present in previous C-fiber models or were shown, in experimental characterization, to be present in peripheral C-fibers (Pelot et al. 2021).*”

10) Page 5, line 85 : How were the channel models validated? This goes to major comment #6 above. What is meant by “basis functions”?

We developed the ion channel models by fitting the steady-state gating values and dynamics to experimental data characterizing changes in gating parameter open probabilities and time constants to step changes in voltage. Because the ion channel models were fit to the experimental data by minimizing the differences between each data point and a standard equation, there is internal validation of the ion channel models. There is also system-level validation because we successfully implemented C-fiber models that reproduced the conduction response data.

Currently, there are not sufficient data available to validate further the models retrospectively. Therefore, prospective experiments need to be conducted to validate the ion channel models’ fidelity. These validations could come in the form of gating parameters and time constants at different voltage levels and at different temperatures.

Basis functions are a mathematical term for the fundamental building blocks that describe a complex system. In our case, the basis functions are the ion channel models because they combine to create a more complex axon model. We clarified our use of this term in the Methods:

*“The ion channel models constituted basis functions, fundamental building blocks that can combine to describe a complex system, for our C-fiber models.”*

We edited the Discussion to emphasize that as additional data become available, we can improve our ion channel models:

“*Nevertheless, we developed a repeatable method to fit a generic equation to patch clamp data from single ion channel characterization, which can be used to create other isoform-specific models and improve our ion channel models as additional data become available.*”

11) Page 8, line 108: What is meant by simulating 50 and 20 PSOs? Does this refer to different sets of initial conditions? If so, why different values for the two types of axons?

We re-ran the PSO 50 (autonomic) and 20 (cutaneous) times with different randomly set initial conditions. We ran a different number of PSOs for the autonomic and cutaneous axons as required to identify a set of membrane parameters that reproduced experimental conduction responses. We clarified these processes in the Results:

“*Thirteen of* *50 autonomic and 14 of 20 cutaneous PSOs identified a set of model parameters that reproduced all the target experimental responses. To evaluate further the 13 autonomic and 14 cutaneous candidate fiber models, we simulated their activity-dependent slowing responses (ADS); one autonomic and one cutaneous fiber model reproduced experimental ADS (Table* *2).*”

12) Page 10, Figure 3, Panels A & B: The authors should comment on why values for conduction velocity and action potential duration determined by PSO did not account for the full range of experimentally-recorded values.

We believe that the PSO-defined C-fiber models did not account for the full range of experimentally recorded conduction velocity and action potential duration values because there may be different types and diameters of autonomic and cutaneous C-fibers with slightly different ion channel densities. This means that different populations of C-fibers are represented in different parts of the range of experimentally recorded conduction responses. This notion of different types of autonomic and cutaneous C-fibers coexisting within a nerve is supported by experimental data showing that there are axons with low threshold, high threshold, and very high threshold responses to activity-dependent slowing stimulation present within the same nerve (Obreja et al. 2010).

We edited the Discussion to include this as a limitation and emphasize that a population of C-fiber models may be needed to model adequately the full range of biological variability.

“*Finally, the PSO-defined C-fiber models, across a range of fiber diameters, did not always account for the full range of experimentally recorded conduction responses. C-fibers with different ion channel densities likely account for the biological variability seen in experimental conduction responses. Thus, future work could use our PSO approach to define a population of autonomic and cutaneous C-fiber models that account for the full range of biological variability.*”

13) Page 14, line 193: Suggest renaming the heading to something like “Specific Ion channels necessary to model C-fiber electrogenesis”

We changed the heading to “Specific Ion Channels are Necessary to Model C-Fiber Conduction Responses.”

14) Page 18, line 253: The authors state that the 12 channel models “accurately represent the experimental patch clamp data”. It appears that in Figures S1-11 the models deviate from the experimentally-recorded values in a number of cases.

We modeled the ion channels by fitting the data to standard equations, and therefore our ion channel models were the best possible representation of the experimental patch clamp data that could be captured by those functional forms. In some cases, such as the fast activation subunit of the NaV1.7 channel, there was quite a bit of variability in the experimental data, and our fit was centered in the experimental data, yet some data points were seemingly far from our ion channel’s dynamics. Therefore, we softened our claim in this sentence:

*“We created a library of 12 isoform-specific ion channel models that ~~accurately~~ represent the available experimental patch clamp data.”*

15) Page 22, line 360: Might be useful to report the conductances/current density for the balance channel/pump as it reports membrane resistivity.

We agree and added the balance conductances and current densities to Table 2.
